# ksrMKL: a novel method for identification of kinase–substrate relationships using multiple kernel learning

**DOI:** 10.7717/peerj.4182

**Published:** 2017-12-20

**Authors:** Minghui Wang, Tao Wang, Ao Li

**Affiliations:** 1 School of Information Science and Technology, University of Science and Technology of China, Hefei, China; 2 Centers for Biomedical Engineering, University of Science and Technology of China, Hefei, China

**Keywords:** ksrMKL, Multiple kernel learning, Kinase identification, Phosphorylation, Kinase–substrate relationships

## Abstract

Phosphorylation exerts a crucial role in multiple biological cellular processes which is catalyzed by protein kinases and closely related to many diseases. Identification of kinase–substrate relationships is important for understanding phosphorylation and provides a fundamental basis for further disease-related research and drug design. In this study, we develop a novel computational method to identify kinase–substrate relationships based on multiple kernel learning. The comparative analysis is based on a 10-fold cross-validation process and the dataset collected from the Phospho.ELM database. The results show that ksrMKL is greatly improved in various measures when compared with the single kernel support vector machine. Furthermore, with an independent test dataset extracted from the PhosphoSitePlus database, we compare ksrMKL with two existing kinase–substrate relationship prediction tools, namely iGPS and PKIS. The experimental results show that ksrMKL has better prediction performance than these existing tools.

## Introduction

As one of the most essential and widespread post-translational modifications in eukaryotes, phosphorylation exerts a crucial role in multiple biological cellular processes which includes regulation of metabolism, DNA repair, gene expression, membrane transport and cellular differentiation (Hunter, 2000; Schlessinger, 2000; Trost & Kusalik, 2011). Phosphorylation activities are catalyzed by protein kinases that regulate a variety of cellular processes, most of which are related to diseases (Hunter, 2000; Manning et al., 2002; Trost & Kusalik, 2013; Zhou et al., 2004). Recent studies (Sharma et al., 2014) show that more than 70% of all proteins (substrates) in human can be catalyzed by protein kinases. Moreover, abnormal activity of protein kinases often causes disease by altering the phosphorylation of substrate proteins, especially in cancer, where protein kinases regulate various cellular processes including movement, apoptosis and cell growth (Bajpai, 2009; Manning et al., 2002; Singh et al., 2005). Therefore, identification of protein kinases responsible for phosphorylation contributes to a better understanding of potential molecular mechanisms and provides a fundamental basis for further disease-related research and drug design.

Therefore, various experimental approaches including low-throughput (Lin et al., 2003; Salinas et al., 2004) and high-throughput (Han et al., 2010; Song et al., 2009; Villén et al., 2007) biological techniques have been developed to identify kinase–substrate relationships. However, low-throughput experimental approaches identify relationships one-by-one manner, resulting in an expensive, time-consuming and labor-intensive process. In contrast, high-throughput biological techniques (e.g., high-throughput mass spectrometry (Villén et al., 2007)) can detect thousands of phosphorylation sites in a single experiment (Han et al., 2010; Song et al., 2009), but cannot provide the corresponding kinase information regarding phosphorylation sites. Considering that the number of newly discovered phosphorylation sites has been exponentially increasing, the huge gap between verified sites and limited kinase information hampers studies of phosphorylation mechanisms as well as the regulatory role of kinases in cellular processes. As a result, the development of new computational methods is required to be developed to help biologists in selecting target kinases and designing related experiments.

Over the past few decades, a variety of computational methods for the identification of kinase–substrate relationships have been developed, most of which build predictive models using local sequence information as kinase catalysis usually occur on the target protein with a specific yet conserved motif (Miller & Blom, 2009). For example, Song et al. (2012) develop a software package, namely iGPS, which employs the predictor in GPS 2.0 (Xue et al., 2008) with local sequence information to discover protein kinases targeting experimentally identified phosphorylation sites. In the meanwhile, by encoding the local sequence of a phosphorylation site with the composition monomer spectrum, Zou et al. (2013) develop a computational tool, namely PKIS, to identify protein kinases for known phosphorylation sites. In addition to the above methods, the recent use of substrate functional information to predict kinase–substrate relationships is gaining increasing attention. For instance, to improve the performance Xu et al. (2014) propose a two-step feature selection algorithm, which takes substrate structure information and high dimensional protein–protein interactions as input. In addition, Linding et al. (2008) develop an online service called NetworKIN that identified kinases using sequence similarity derived from Scansite and NetPhosK, and a probabilistic network of functional associations extracted from the STRING (Szklarczyk et al., 2011) database.

Inspired by the above methods, we put forward a novel computational method, namely ksrMKL, based on multiple kernel learning (MKL) (Gönen & Alpaydın, 2011) for identifying kinase–substrate relationships. The proposed method takes advantage of not only sequence information but also functional information regarding substrates that are reported to contribute to phosphorylation site prediction (Gnad et al., 2007; Huang et al., 2005; Iakoucheva et al., 2004). To efficiently utilize local sequence information and functional information, we develop multiple kernels using the radial basis function (RBF) (Scholkopf et al., 1997) as a kernel. Subsequently, we use MKL to combine multiple kernels and build a support vector machine (SVM) model using the combined kernel. The comparative analysis is based on 10-fold cross-validation process and the collected data from the Phospho.ELM (Dinkel et al., 2010) database. The experimental results show that ksrMKL is greatly improved in various measures compared with a single kernel SVM (Chang & Lin, 2011; Wang, Jiang & Xu, 2015). Furthermore, with an independent test dataset extracted from the PhosphoSitePlus (Hornbeck et al., 2012) database, we compare ksrMKL with two existing kinase–substrate relationship prediction tools, namely iGPS (Song et al., 2012) and PKIS (Zou et al., 2013). The results show that ksrMKL has better prediction performance than these existing tools.

## Materials and Methods

### Data collection and preparation

In this study, we adopt an experimental identification of phosphorylation sites in human with kinase information dataset, including 1,638 unique phosphorylation sites in 679 substrates collected from the latest version of Phospho.ELM (Dinkel et al., 2010). Blastclust (Dondoshansky & Wolf, 2002) with a 70% threshold is used for this dataset to avoid protein redundancy and homology (Xu et al., 2014). In terms of a specific kinase, the phosphorylation sites that are known to be modified by this kinase are considered as positive samples, and the phosphorylation sites that are not known to be modified by this kinase are used as negative samples. To ensure reliable results (Li et al., 2015; Xue et al., 2011), we analyze kinases that contain not less than 25 positive phosphorylation sites and eventually 17 kinases are obtained. The detailed information of this dataset is summarized in Table S1. Besides, local sequences of the corresponding phosphorylation sites are also extracted containing seven residues upstream and seven residues downstream. In this study, we follow the procedure described in (Wang, Jiang & Xu, 2015) and use binary encoding to convert each amino acid of local sequence into a 21-dimensional binary vector. The 15-length local sequence is converted to a 315-dimensional vector. In addition, several recent studies (Fan et al., 2014; Li, Du & Xu, 2010; Xu et al., 2014) have shown that protein (substrate) function information (e.g., PPI information) can effectively improve the prediction performance for kinase–substrate relationships. By following these studies, we incorporate PPI information as functional information of substrate into the proposed method. Here, the PPI information is extracted from human data of STRING (Szklarczyk et al., 2011) database. Finally, 16,708 proteins that interacted with the 679 substrates are obtained. The functional information that are employed as a 16,708-length feature vectors and the local sequence using binary encoding are incorporated to generate the final feature vectors.

### Multiple kernel learning

Recently, MKL has been widely applied in the field of bioinformatics (Brayet et al., 2014; Nascimento, Prudêncio & Costa, 2016; Shen et al., 2014) (e.g., drug–target interaction prediction (Nascimento, Prudêncio & Costa, 2016)), which can be used to combine different data types with different measurements or sources. The use of multiple kernels instead of a single kernel makes the decision function more interpretable and improves performance (Rakotomamonjy, Bach & Grandvalet, 2007; Zhang et al., 2016). The linear combination of multiple kernel is defined as follows:

(1) $$\matrix{{{K_{\rm{\eta }}} = \mathop \sum \limits_{r = 1}^R {{\rm{\eta }}_r}{K_r},} & {{{\rm{\eta }}_r} \ge 0}  \cr } $$

where η_*r*_ denotes the kernel weight and *K_r_* is the *r’th* basic kernel. To obtain kernel weights, various methods have been extensively studied (Aiolli & Donini, 2014, 2015; Gönen & Alpaydın, 2011), which can be roughly divided into fixed or heuristic-based methods and optimization-based methods. Regarding fixed or heuristic-based methods, the combination is obtained by using fixed rules, while its effectiveness crucially hinges on the domain at hand (Aiolli & Donini, 2015; Gönen & Alpaydın, 2011). For optimization-based methods, the combination parameters are achieved by solving an optimization problem formulated as a different model or directly integrated into the learning machine (Aiolli & Donini, 2015; Gönen & Alpaydın, 2011).

In this study, we employ an optimization-based method proposed by Aiolli and Donini, namely EasyMKL (Aiolli & Donini, 2014, 2015), which maximizes the distance between the convex hulls of positive and negative samples on the training set (Donini et al., 2016). In EasyMKL, the combination parameters are obtained by solving the following formula:

(2) $$\mathop {{\rm{max}}}\limits_{ \eta = 1 } \mathop {{\rm{min}}}\limits_{\gamma \in \Gamma } {\gamma ^T}Y\left( {\mathop {\mathop \sum \nolimits^ }\limits_{r = 0}^R {\eta _r}{K_r}} \right)Y\gamma + \lambda  \gamma { ^2}$$

where λ is a regularization parameter, and *Y* is a diagonal matrix of training labels. The domain Γ represents two probability distributions of the positive and negative samples, defined as ${\rm{\Gamma }}\;{\rm{ = }}\;\left\{ {{\rm{\gamma }}\; \in \;R_ + ^1|\sum\nolimits_{{y_i} = + 1} {{{\rm{\gamma }}_i} = 1,} \sum\nolimits_{{y_i} =-1} {{{\rm{\gamma }}_i}} = 1} \right\}$. The objective function can be converted into a regularized empirical dual problem with the kernel $\mathop \sum \nolimits_{r = 1}^R {{\rm{\eta }}_r}{K_r}$. With the derivation described in Aiolli & Donini (2015), this minimax problem can be simplified to a quadratic problem. The optimal solution γ* of the quadratic problem is equivalent to the solution of the original min–max formulation. According to the structure of EasyMKL (Donini et al., 2016), the average kernel of all the trace-normalized basic kernels (${K^A} = {1 \over R}\mathop \sum \nolimits_{r = 1}^R {{{K_r}} \over {{T_r}\left( {{K_r}} \right)}}$) can also be obtained. Taking the optimal solution γ* and the average kernel, the optimal weight for a single basic kernel *K_r_* is achieved through the following formula:

(3) $$\matrix{ {{{\rm{\eta }}_r} = {{\rm{\gamma }}^{*T}}Y\left({{K_r}/{T_r}\left({{K_r}} \right)} \right)Y{{\rm{\gamma }}^*},} & {\forall r = 1, \cdots, R} \cr } $$

After obtaining the combined kernel using Eqs. (2) and (3), the SVM algorithm is used to build predictive models and the decision function is defined as follows:

(4) $$\matrix{   {\mathop {\min }\limits_{f,b,{\rm{\xi }}} \quad {1 \over 2}\left\| f \right\|_{\rm{H}}^2 + C\mathop {\mathop \sum \nolimits^ }\limits_i {{\rm{\xi }}_i}} \hfill  \cr    {{\rm{s}}{\rm{.t}}{\rm{.}}\quad {y_i}\left( {f\left( {{x_i}} \right) + b} \right) \ge 1 - {{\rm{\xi }}_i}\quad \forall i} \hfill  \cr    {\quad \quad \quad \quad \quad \quad \;\,{{\rm{\xi }}_i} \ge 0\quad \forall i} \hfill  \cr  } $$

where ‖*f*‖_H_ denotes the kernel in Hilbert space, which is associated with the kernel *K*_η_. Therefore, in terms of the kernel function, the discriminant function takes the following form:

(5) $$f\left(x \right) = \mathop \sum \limits_{i = 1}^n {\rm{\alpha }}_i^*{k_{\rm{\eta }}}\left({x,{x_i}} \right) + {b^*}$$

### Implementation procedures of the proposed system

The input features are divided into two different data types (including local sequence and functional information) based on their original types. Afterward, we follow previous studies (Hasan, Ahmad & Molla, 2017; Zhang et al., 2016) and utilize the RBF kernel function with multiple beta values to computer the base kernels for each data type, and the RBF kernel is defined according to $K\left({{x_i},{x_j}} \right) = {\rm{exp}}\left\{ {-{{\rm{\beta }} \over {\left| {{F_{\rm{n}}}} \right|}}||{x_i}-{x_j}|{|^2}} \right\}$, where |*F*_n_| is the number of features. The whole implementation of our method is summarized as follows:

1. Discretizing the parameter space of the beta (β) of the RBF kernel into five values to obtain the set of the base kernels $S = \left\{ {{K_{{{\rm{\beta }}_1}}},{K_{{{\rm{\beta }}_2}}}, \cdots, {K_{{{\rm{\beta }}_5}}}} \right\}$ for each data type. In this study, five values of beta for the base kernels are {1, 2,…, 5}, and finally 10 kernels are obtained through two different data types.
2. Finding kernel weights for these 10 kernels using Eq. (3).
3. Combining these 10 kernels using Eq. (1) to obtain the combined kernel.
4. Using the combined kernel to train the predictive model for each kinase.
5. Using these models to make predictions for a potential phosphorylation site.

### Performance evaluation

In this study, by following existing studies (Gao et al., 2010; Wang, Wang & Li, 2017), the 10-fold cross-validation is implemented on the known experimentally verified substrate–kinase relationships dataset, in which the dataset is divided into ten parts, followed by iteratively taking nine as training data and the remaining one as test data until all parts have been tested. The receiver-operating characteristic (ROC) curve and the corresponding area under ROC curve (AUC) are used to estimate the predictive ability of the proposed method. Besides, other conventional measurements such as sensitivity (Sn), F-Measure (F1), specificity (Sp), precision (Pre) and Matthews’s correlation coefficient (MCC) are also adopted to assess the predictive performance, defined as follows:

(6) $${\rm{Sn}} = {{{\rm{TP}}} \over {{\rm{FN}} + {\rm{TP}}}}$$

(7) $${\rm{Sp}} = {{{\rm{TN}}} \over {{\rm{TN}} + {\rm{FP}}}}$$

(8) $${\rm{Pre}} = {{{\rm{TP}}} \over {{\rm{FP}} + {\rm{TP}}}}$$

(9) $${\rm{F}}1 = {{2 \times {\rm{Pre}} \times {\rm{Sn}}} \over {{\rm{Pre}} + {\rm{Sn}}}}$$

(10) $${\rm{MCC}} = {{{\rm{TP}} \times {\rm{TN}}-{\rm{FP}} \times {\rm{FN}}} \over {\sqrt {\left({{\rm{TN}} + {\rm{FN}}} \right) \times \left({{\rm{TN}} + {\rm{FP}}} \right) \times \left({{\rm{FP}} + {\rm{FN}}} \right) \times \left({{\rm{TP}} + {\rm{FP}}} \right)} }}$$

where TN and TP represent the number of positive and negative sites that are correctly predicted, commonly called true negative and true positive, respectively. FP and FN represent the number of positive and negative sites that are wrong predicted, commonly called false negative and false positive, respectively. It is noteworthy that when the positive and negative sites are significantly imbalanced, MCC can be used to achieve the balance quality.

## Results

### Evaluate performance using 10-fold cross-validation

**Figure 1:**
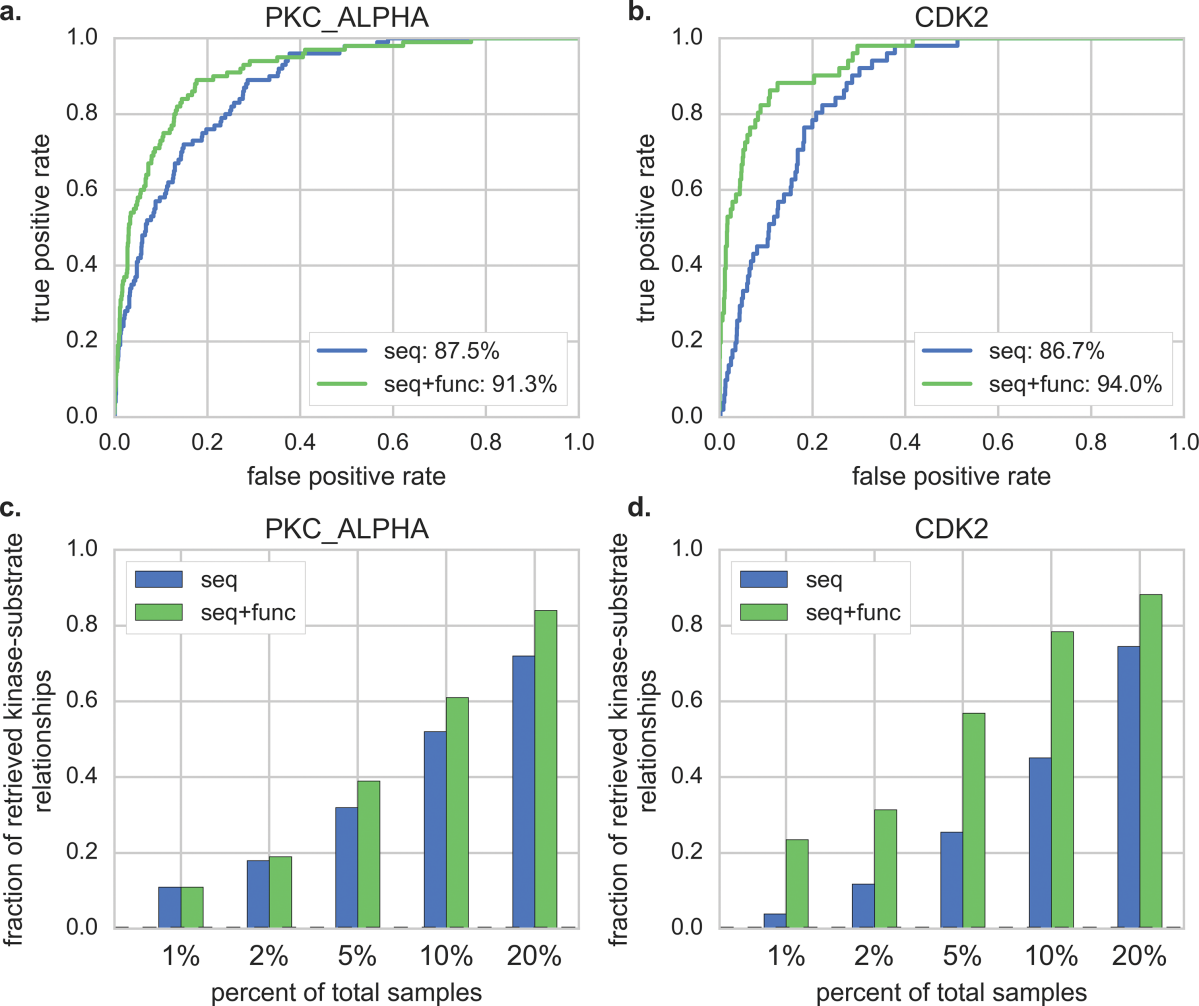
Comparison of ROC curves and the fraction of retrieved kinase–substrate relationships using different information. Panels (A and B) represent the ROC curves of PKC_ALPHA and CDK2 using different information, respectively. Panels (C and D) represent the fraction of retrieved kinase–substrate relationships of PKC_ALPHA and CDK2 using different information, respectively. The blue lines/bars represent our proposed method constructed with local sequence, and the green lines/bars represent our proposed method built with local sequence and functional information together.

To verify the effectiveness of ksrMKL, we firstly compare the prediction performance before and after integrating the functional information. Two kinases are taken as examples to illustrate the predictive performance and the corresponding ROC curves are displayed in Figs. 1A and 1B. As shown in Figs. 1A and 1B, by combining the functional information, ksrMKL achieves better overall performance than using local sequences only. For example, for PKC_ALPHA (Fig. 1A), ksrMKL_seq+func_ achieves an AUC value of 91.3%, and the corresponding AUC value of ksrMKL_seq_ is 87.5%. Similarly, for CDK2 (Fig. 1B), the AUC value obtained by ksrMKL_seq+func_ is also increased by 7.3% in comparison with ksrMKL_seq_. The performance of other kinases is also displayed in Fig. S1. Admittedly, in the field of computational bioinformatics, the ability to control false positive prediction results is usually important (Xu & Wang, 2016). Hence, to verify the controllability, we follow the previous studies (Wang, Wang & Li, 2017; Xu & Wang, 2016) and calculate the true positives number of top-ranked results. Figures 1C and 1D displays the results of five top 1%, 2%, 5%, 10% and 20% of the total samples in PKC_ALPHA (Fig. 1C) and CDK2 (Fig. 1D). Obviously, ksrMKL_seq+func_ can obtain better performance at any percent of the total sample when compared with ksrMKL_seq_. In general, these results indicate that the functional information can effectively enhance the predictive performance of kinase–substrate relationships, and our proposed method can successfully combine different information.

**Figure 2:**
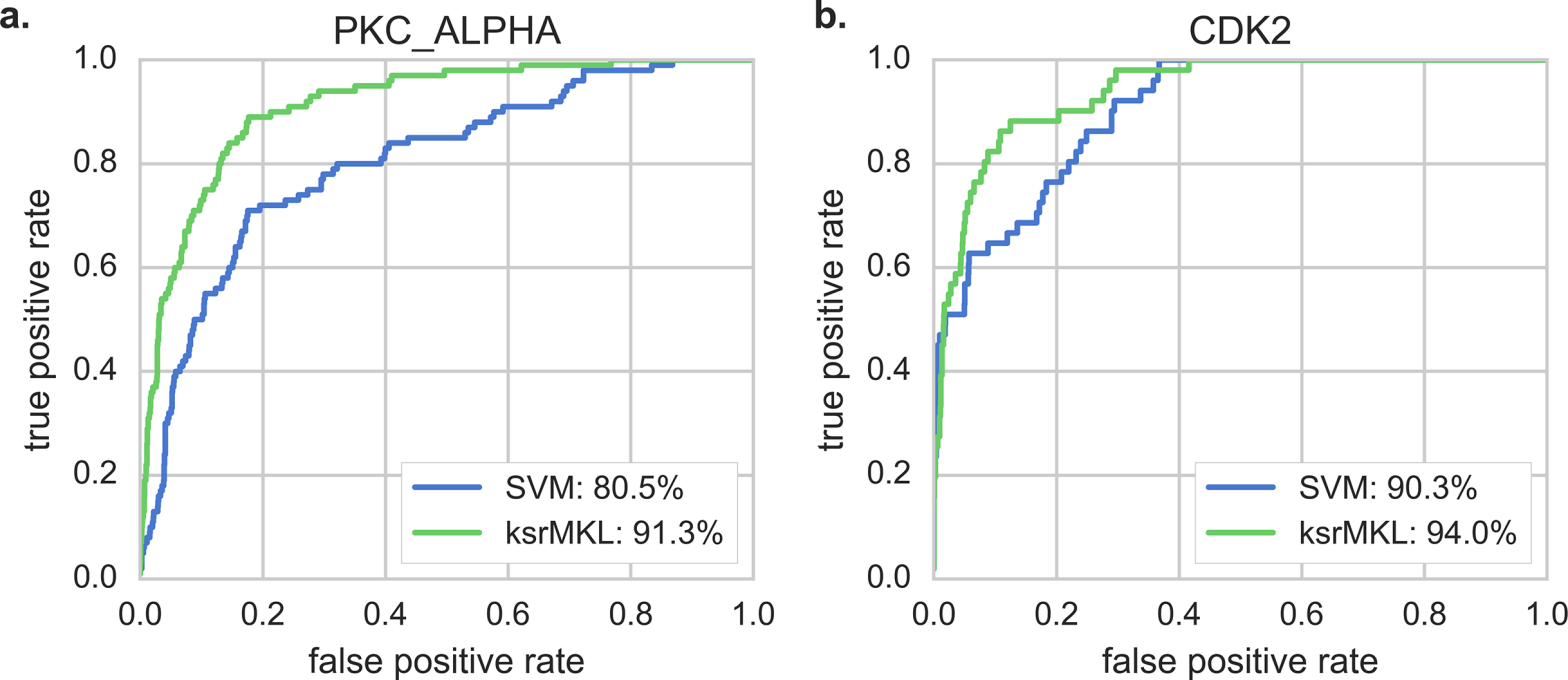
Comparison of ROC curves with different methods. Panels (A and B) represent the ROC curves of PKC_ALPHA and CDK2 using different methods, respectively. The green lines represent our proposed method (ksrMKL) and the blue lines represent SVM method.

Secondly, we make a comparison between ksrMKL and single kernel SVM with the same features. The ROC curves and AUC values obtained using two methods can be found in Fig. 2, indicating that ksrMKL has the highest true positive rate at each false positive rate in PKC_ALPHA (Fig. 2A) and CDK2 (Fig. 2B). For example, for CDK2, the AUC value of ksrMKL is 94.0%, which is higher than that obtained using SVM (90.3%). The performance of other kinases is also displayed in Fig. S2. These results suggest that ksrMKL has a better predictive ability compared with SVM. Additionally, to further verify the effectiveness of ksrMKL, according to previous studies (Fan et al., 2014; Wang, Wang & Li, 2017), we set a threshold for each method so that the specificity of each method is equal to 90.0% (medium) or 95.0% (high). Then, the corresponding measurements are calculated and the results are presented in Table 1. With specificity of 95.0%, all other measurements are higher than that with SVM. When specificity is reduced to 90.0%, the measurement of both methods increases and ksrMKL shows consistently higher performance in all the above measurements compared with SVM. In summary, ksrMKL can achieve better performance in kinase–substrate relationship prediction compared with the conventional single kernel-based SVM method.

**Table 1:** Comparison of predictive performance using different methods at high (Sp = 95.0%) and medium (Sp = 90.0%) stringency level.

Kinases	Methods	Sp = 95%				Sp = 90%			
		Sn (%)	MCC (%)	F1 (%)	Pre (%)	Sn (%)	MCC (%)	F1 (%)	Pre (%)
GSK3b	SVM	43.9	25.6	25.9	18.4	48.8	19.4	18.1	11.1
	ksrMKL	56.1	32.9	31.9	22.3	65.9	27.4	23.7	14.4
CDK2	SVM	51.0	32.6	33.3	24.8	64.7	29.5	27.2	17.2
	ksrMKL	66.7	42.3	41.5	30.1	82.4	38.3	33.3	20.9
Lck	SVM	57.8	35.1	34.4	24.5	77.8	34.3	29.3	18.0
	ksrMKL	60.0	36.4	35.5	25.2	80.0	35.3	30.0	18.5
EGFR	SVM	55.6	31.0	29.4	20.0	66.7	26.3	21.8	13.0
	ksrMKL	58.3	32.5	30.7	20.8	77.8	31.2	25.0	14.9
Abl	SVM	50.0	25.8	24.0	15.8	63.3	22.9	18.1	10.6
	ksrMKL	66.7	34.6	30.8	20.0	76.7	28.3	21.5	12.5
PKCa	SVM	32.0	25.9	30.6	29.4	50.0	29.0	32.9	24.5
	ksrMKL	57.0	45.4	48.7	42.5	73.0	43.6	44.6	32.2
Fyn	SVM	60.0	31.1	28.1	18.4	70.0	25.6	19.8	11.5
	ksrMKL	66.7	34.6	30.8	20.0	86.7	32.3	24.0	13.9
ATM	SVM	62.7	39.9	39.5	28.8	70.6	32.5	29.3	18.5
	ksrMKL	94.1	57.9	53.9	37.8	98.0	45.8	38.5	23.9

### Comparison with existing kinase–substrate relationship tools

**Figure 3:**
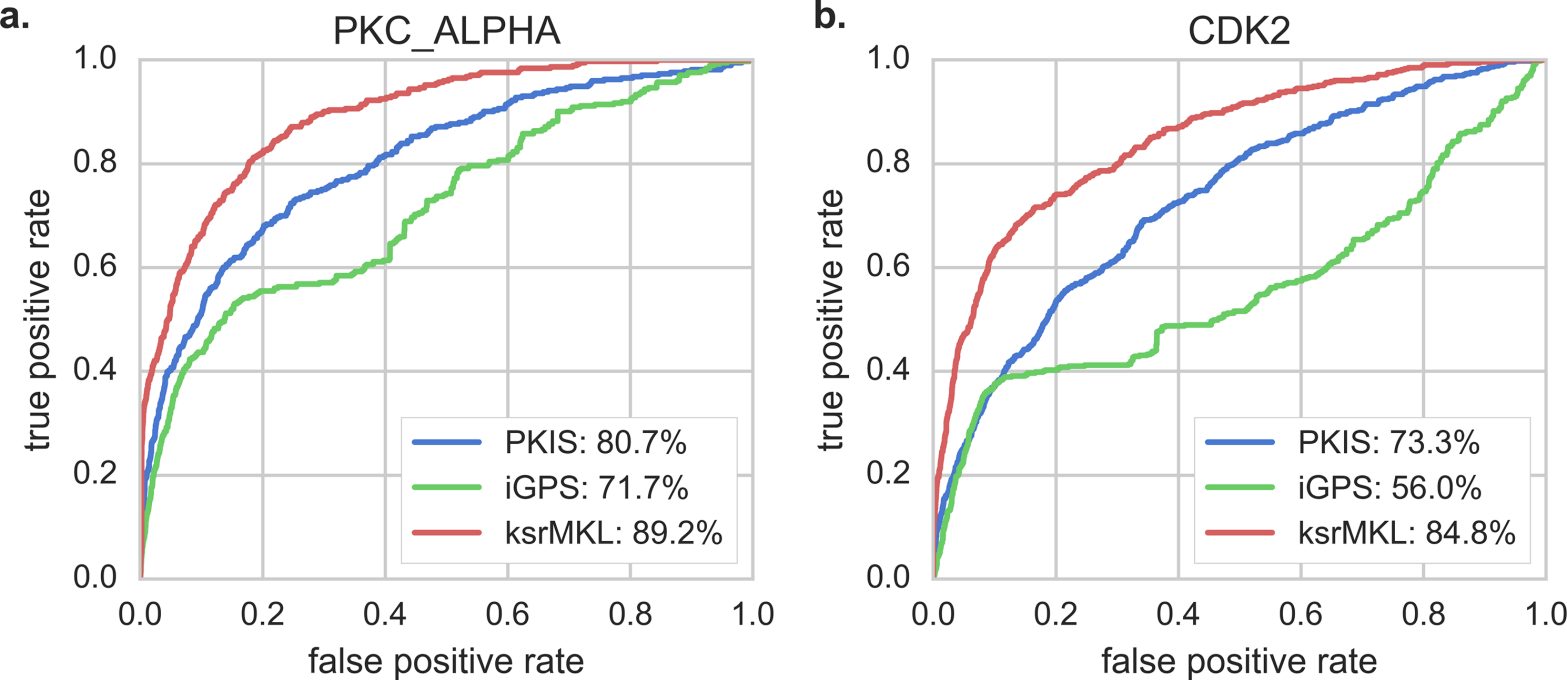
Comparison of ROC curves with existing tools on the independent dataset. Panels (A and B) represent the ROC curves of PKC_ALPHA and CDK2 using different tools on the independent dataset, respectively. The red lines represent our proposed method (ksrMKL), and the green and blue lines represent iGPS and PKIS tools, respectively.

In this section, we compare ksrMKL with two common kinase–substrate relationship prediction tools, namely iGPS (Song et al., 2012) and PKIS (Zou et al., 2013), to further verify the advantages of our method. Since iGPS and PKIS use the dataset extracted from Phospho.ELM database to build prediction models, the dataset should at least be divided into training and test datasets, which will inevitably result in over-estimation of prediction performance (Xu et al., 2014). To solve this problem, we build an independent test dataset from the latest PhosphoSitePlus (Hornbeck et al., 2012) database, which excludes the existing phosphorylation sites deposited in Phospho.ELM (9.0). We take the above two kinases as examples to demonstrate predictive performance and the results can be found in Fig. 3. As shown in Fig. 3, compared with other methods, ksrMKL achieves a better overall performance in PKC_ALPHA (Fig. 3A) and CDK2 (Fig. 3B). For example, for PKC_ALPHA, the AUC achieved by ksrMKL is 17.5% and 8.5% higher than iGPS and PKIS, respectively. Likewise, for CDK2, the corresponding AUC values are 84.8%, 56.0% and 73.3% for ksrMKL, iGPS and PKIS, respectively. Figure S3 displays the performance of other kinases.

**Figure 4:**
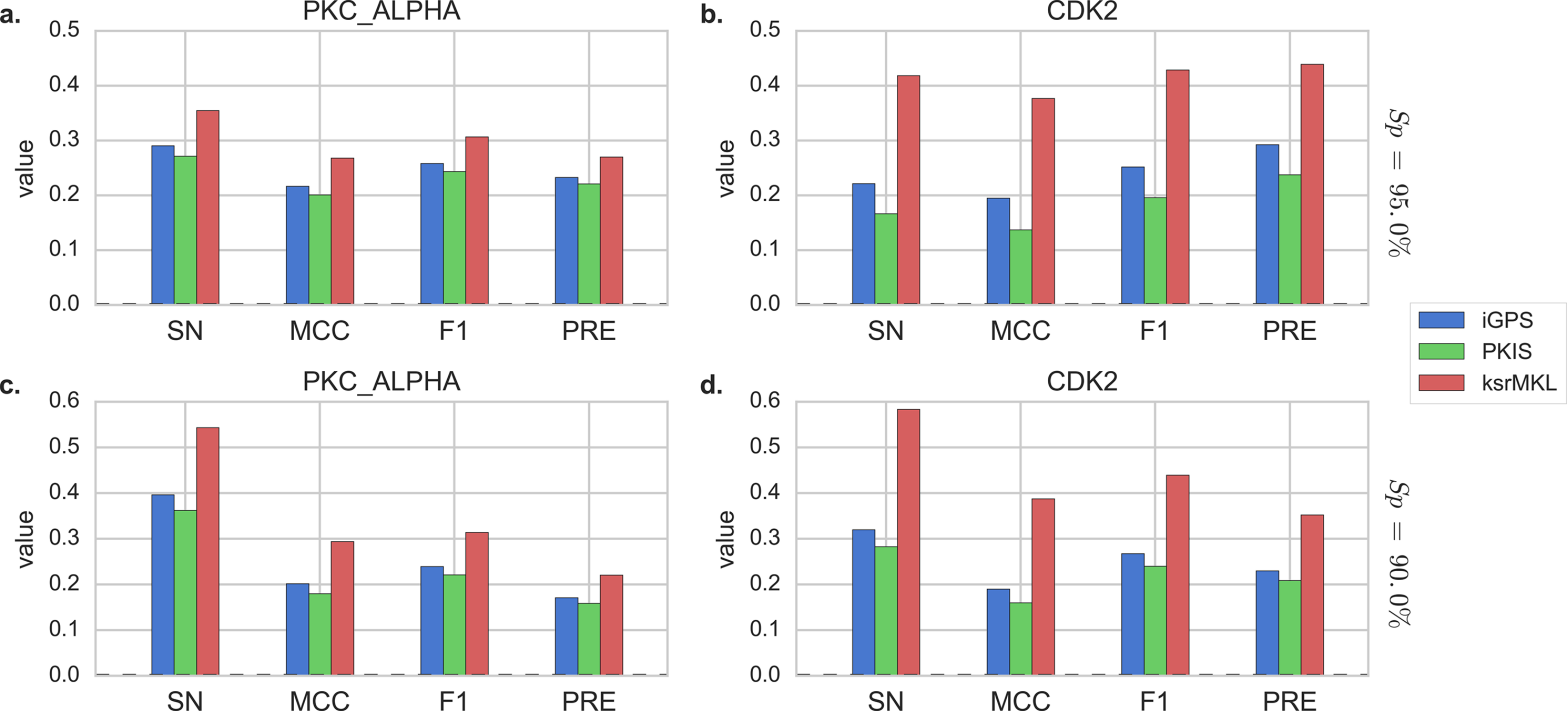
Comparison of Sn, MCC, F1 and Pre values of different tools on the independent dataset. Panels (A and B) represent the performance of PKC_ALPHA and CDK2 at specificity of 95.0%, and Panels (C and D) represent the performance of PKC_ALPHA and CDK2 at specificity of 90.0%. The *x*-axis represents sensitivity, Matthew correlation coefficient, F1-measure and precision, respectively.

**Figure 5:**
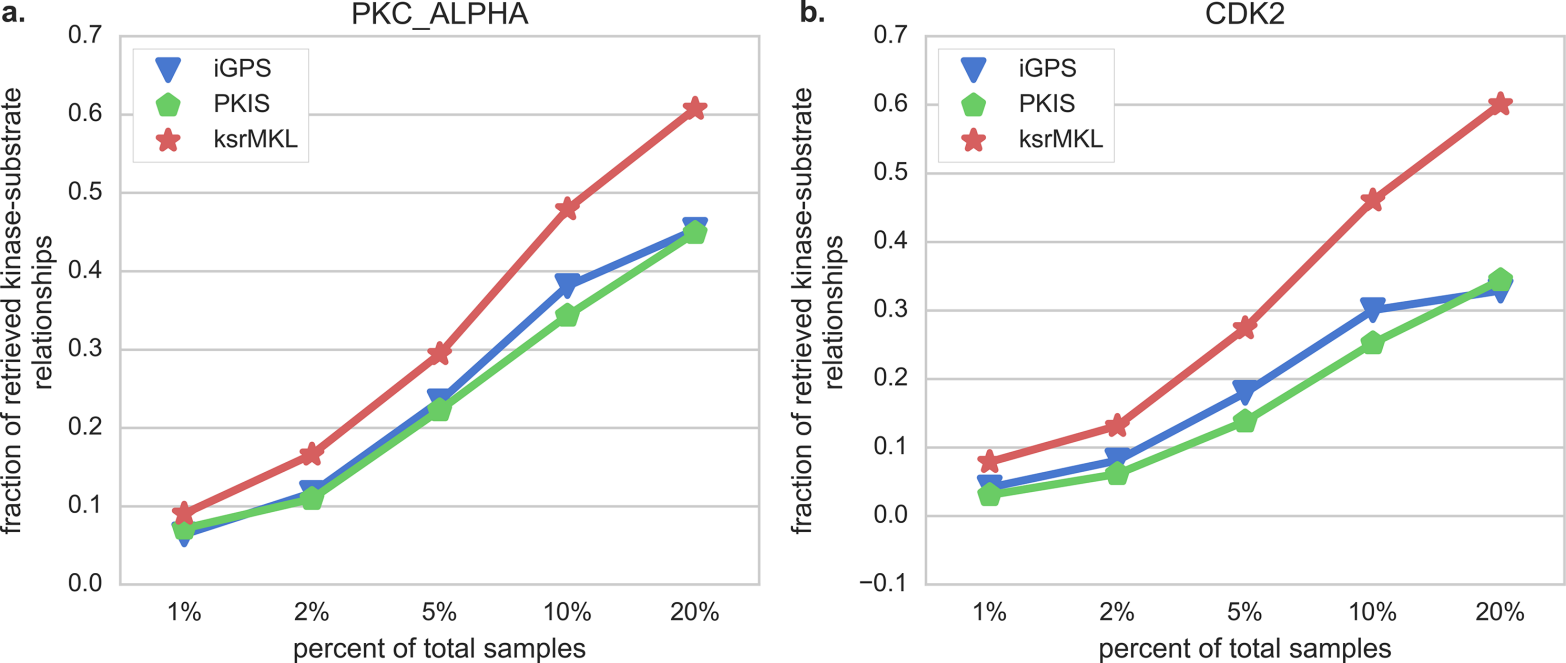
Comparison of the ability of different tools in retrieve kinase–substrate relationships. Panels (A and B) represent the fraction of retrieved kinase–substrate relationships of PKC_ALPHA and CDK2 using different tools on the independent dataset, respectively. The red lines represent our proposed method (ksrMKL), and the green and blue lines represent iGPS and PKIS tools, respectively.

Additionally, the comparisons of Sn, MCC, Pre and F1 with two kinases at the two stringency levels are also drawn on Fig. 4, indicating that in almost all cases ksrMKL achieves the best performance in PKC_ALPHA (Figs. 4A and 4C) and CDK2 (Figs. 4B and 4D). Taking CDK2 as an example, with specificity of 95.0% (Fig. 4B), the Sn, MCC, Pre and F1 values of ksrMKL are increased by 19.7%, 18.2%, 17.7% and 14.6% compared with iGPS and make an improvement of 25.2%, 23.9%, 23.3% and 20.2% when compared with PKIS, respectively. When the specificity is reduced to 90.0% (Fig. 4D), ksrMKL has an improvement of 26.3%, 19.8%, 17.2% and 12.2% compared with iGPS. Likewise, in comparison with PKIS, the Sn, MCC, Pre and F1 values are increased by 30.0%, 22.8%, 19.9% and 14.4%, respectively. Table S2 lists the results for other kinases. According to Table S2, at the high stringency level, the Sn, MCC, F1 and Pre values of ksrMKL on average are increased by 12.1%, 7.2%, 6.0% and 4.1% compared with iGPS and have an improvement of 22.6%, 12.5%, 10.3% and 7.2% in comparison with PKIS, respectively. Furthermore, the controllability of false positive prediction results is also employed to estimate the predictive performance of these three methods. Figure 5 shows the results of five top 1%, 2%, 5%, 10% and 20% of the total samples. ksrMKL makes most of the known sites higher ranks than other tools investigated in this study. For example, for PKC_ALPHA (Fig. 5A), at the top 20% the proposed method achieves a fraction of true positives of 60.8% and the values of iGPS and PKIS are 45.3% and 44.9%, respectively. Similarly, for CDK2 (Fig. 5B), we can obtain similar results. In summary, the aforementioned analysis suggest that ksrMKL improves the prediction of kinase–substrate relationships when compared with existing tools.

### Analysis of the predicted potential relationships

In the above section, we have validated that ksrMKL has good prediction performance in kinase–substrate relationships. However, due to the difficulty of experimental verification, the computational method requires the ability to detect unknown relationships between phosphorylation site and protein kinase (Xu & Wang, 2016). Consequently, we analyze the top 20 ranked candidate phosphorylation sites that are not modified by a corresponding kinase in our dataset and then try confirming these results by mining the literature and searching the UniProtKB database. Table 2 displays the detailed top 20 potential phosphorylation sites of CDK2 and the related information of substrates. We find some phosphorylation sites have been demonstrated to be modified by CDK2. For example, from the UniProtKB database, it can be found that the phosphorylation site Ser640, Ser964 and Ser975 of RBL1 can be modified by CDK2 (http://www.uniprot.org/uniprot/P28749#ptm_processing). Furthermore, in Table S3, we also list the top 20 ranked potential phosphorylation sites for MAPK1, in which Tyr325 and Tyr331 of FOS (P01100) has been confirmed to be modified by this kinase (http://www.uniprot.org/uniprot/P01100#ptm_processing). These results demonstrate that ksrMKL has the ability to discover potential kinase–substrate relationships, which could be conducive to further experimental verification.

**Table 2:** Information of top 20 potential phosphorylation sites for CDK2 kinase.

Ranking	UniProt ID	Protein name	Position	Score	Ranking	UniProt ID	Protein name	Position	Score
1	P28749	RBL1	369	0.909	11	P28749	RBL1	964	0.542
2	Q08999	RBL2	401	0.750	12	Q08999	RBL2	672	0.542
3	P28749	RBL1	975	0.742	13	P17480	UBTF	201	0.505
4	Q08999	RBL2	1035	0.682	14	P38936	CDKN1A	98	0.494
5	P28749	RBL1	640	0.617	15	P38398	BRCA1	988	0.459
6	P17480	UBTF	117	0.577	16	P38936	CDKN1A	57	0.418
7	P46527	CDKN1B	178	0.570	17	Q13415	ORC1	273	0.412
8	Q13415	ORC1	258	0.564	18	P17480	UBTF	484	0.362
9	Q15796	SMAD2	8	0.559	19	P31350	RRM2	20	0.266
10	P46527	CDKN1B	10	0.558	20	P06401	PGR	294	0.259

## Discussion and Conclusion

Phosphorylation exerts a crucial role in multiple biological cellular processes which is catalyzed by protein kinases and closely related to many diseases. Therefore, identification of potential protein kinases for experimentally verified phosphorylation sites is important for understanding molecular mechanisms and provides a fundamental basis for further disease-related research and drug design. Considering the labor-intensiveness and high cost of experimental identification, efficient and rapid protein kinase identification computational methods are urgently needed. Accordingly, we develop a computational method to identify protein kinases based on MKL. Under a 10-fold cross-validation process and an independent test dataset, ksrMKL has better prediction performance than existing computational tools including single kernel SVM, which indicates that MKL could be very useful for the identification of protein kinases. Furthermore, through the analysis of the predicted potential kinase modified phosphorylation sites, we find that some highly ranked results have been confirmed in the UniProtKB database, which suggest that ksrMKL can be used to discover potential protein kinases for experimentally verified phosphorylation sites and further help subsequent experimental verification.

The improvement of ksrMKL relative to other methods could be attributed to two factors. First, we take advantage of sequence information as well as functional information of substrates to construct a predictive model. Second, in our proposed method, different kernels use input coming from different heterogeneous information sources and combining these kernels would increase the generalization of the model. Although ksrMKL exhibits excellent performance in kinase identification, it can be further improved from various perspectives. For instance, other biological information (e.g., structure information of substrates) could be incorporated to further improve performance. In addition, the functional information used in this study is extracted from the STRING (Szklarczyk et al., 2011) database, and there are many other related databases (e.g., MINT (Licata et al., 2012)), which can be included to further improve the performance of our proposed method. Moreover, more experimentally verified phosphorylation sites with associated kinase information deposited in other bioinformatics resources can be combined to build better prediction models, as more training data usually improves classification performance.

## Supplemental Information

10.7717/peerj.4182/supp-1 Supplemental Information 1 Supplementary material.

10.7717/peerj.4182/supp-2 Supplemental Information 2 Code and data.

## Additional Information and Declarations

### Data Availability

The following information was supplied regarding data availability: The raw data and code has been supplied as Supplemental Dataset Files.
