# Supplementary material for: ksrMKL: a novel method for identification of kinase–substrate relationships using multiple kernel learning
Source: PeerJ. 2017 Dec 20;5:e4182. doi: 10.7717/peerj.4182 (PMC5741978; doi:10.7717/peerj.4182)
Supplement: Supplemental Information 1 [file peerj-05-4182-s001.pdf]

Supplementary Material

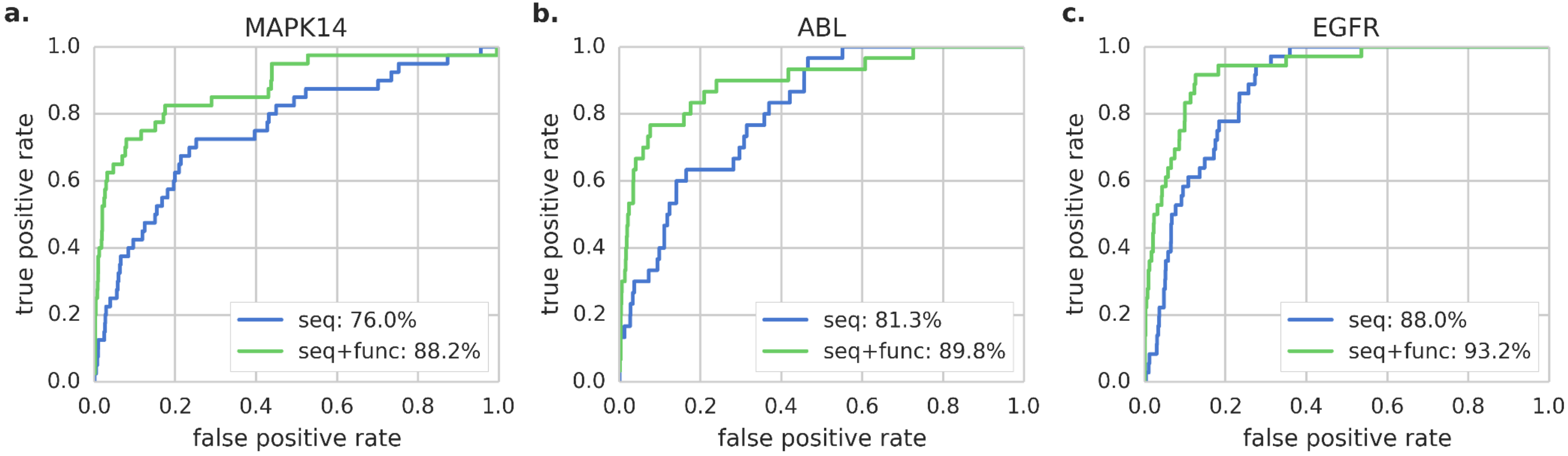

Figure S1. Comparison of ROC curves using different information. Panels (a-c) represent the ROC curves of MAPK14, ABL and EGFR using different information, respectively. The blue lines represent our proposed method constructed with local sequence, and the green lines represent our proposed method built with local sequence and functional information together.

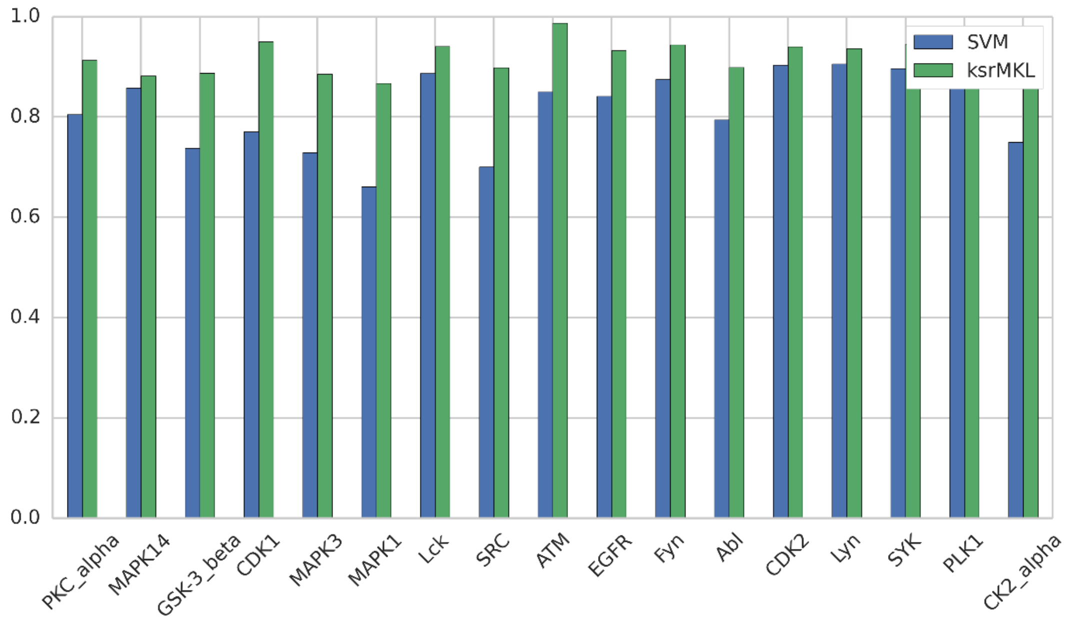

Figure S2. Compare the AUC values with different methods. The green lines represent the ROC curve of ksrMKL, and the blue lines represent the ROC curves of SVM.

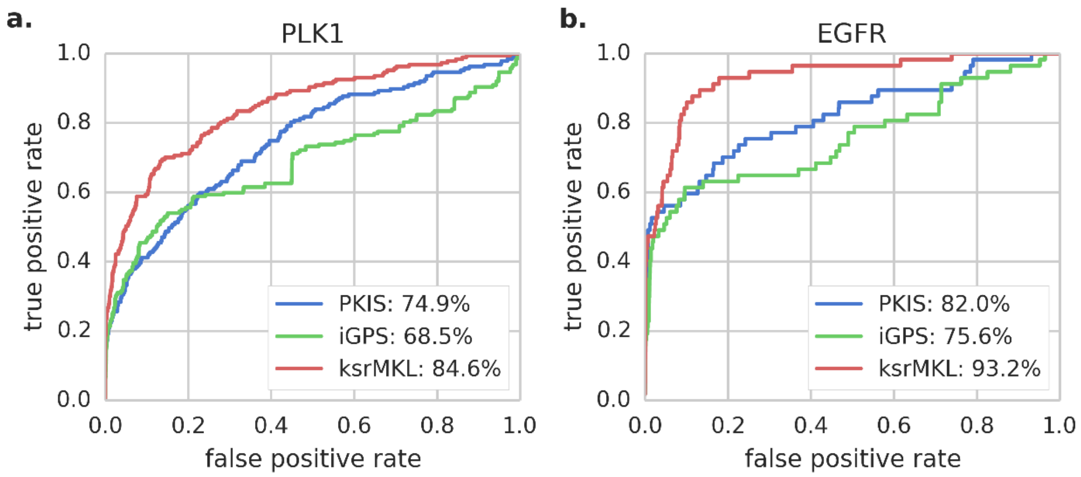

Figure S3. Compare the ROC curves with existing tools on the independent dataset. Panels (a-b) represent the ROC curves of PLK1 and EGFR using different tools on the independent dataset, respectively. The red lines represent our proposed method (ksrMKL), and the green and blue lines represent iGPS and PKIS tools, respectively.

Table S1. The number of known phosphorylation sites of 17 kinases.

Kinases	Number	Kinases	Number
PKCa	100	EGFR	36
MAPK14	40	Fyn	30
GSK3b	41	Abl	30
CDK1	112	CDK2	51
MAPK3	71	Lyn	28
MAPK1	83	SYK	37
Lck	45	PLK1	37
SRC	87	CK2a	99
ATM	51		

Table S2. Compare the predictive performance of different tools at high (Sp = 95.0%) and medium (Sp = 90.0%) stringency level.

Kinase	Method	Sp = 95%				Sp = 90%			
		Sn	MCC	F1	Pre	Sn	MCC	F1	Pre
CK2a	iGPS	50.0%	9.7%	4.2%	2.2%	66.7%	8.9%	2.9%	1.5%
	PKIS	25.0%	4.3%	2.1%	1.1%	58.3%	7.6%	2.5%	1.3%
	ksrMKL	58.3%	11.4%	4.9%	2.6%	83.3%	11.5%	3.6%	1.8%
Lyn	iGPS	18.6%	5.5%	5.1%	2.9%	34.9%	7.3%	5.1%	2.8%
	PKIS	16.3%	4.6%	4.4%	2.6%	27.9%	5.3%	4.1%	2.2%
	ksrMKL	39.5%	13.8%	10.5%	6.0%	58.1%	14.1%	8.4%	4.5%
PLK1	iGPS	27.3%	15.5%	17.4%	12.8%	42.4%	16.6%	16.4%	10.2%
	PKIS	19.4%	10.2%	12.7%	9.4%	30.9%	10.8%	12.2%	7.6%
	ksrMKL	43.2%	25.6%	26.1%	18.8%	56.1%	23.3%	21.1%	13.0%
SYK	iGPS	40.0%	6.9%	2.8%	1.5%	50.0%	5.7%	1.8%	0.9%
	PKIS	30.0%	4.9%	2.1%	1.1%	40.0%	4.3%	1.5%	0.7%
	ksrMKL	40.0%	6.9%	2.8%	1.5%	60.0%	7.2%	2.2%	1.1%
Abl	iGPS	30.8%	14.7%	14.7%	9.6%	30.8%	8.8%	8.7%	5.1%
	PKIS	38.5%	18.9%	18.0%	11.7%	52.7%	17.9%	14.5%	8.4%
	ksrMKL	56.0%	28.1%	25.2%	16.2%	81.3%	29.3%	21.4%	12.4%
CDK2	iGPS	22.1%	19.5%	25.2%	29.3%	32.0%	19.0%	26.8%	23.0%
	PKIS	16.7%	13.7%	19.6%	23.8%	28.3%	16.0%	24.0%	20.9%
	ksrMKL	41.9%	37.7%	42.9%	43.9%	58.3%	38.8%	43.9%	35.2%
EGFR	iGPS	30.0%	8.5%	5.9%	3.3%	36.7%	6.6%	3.8%	2.0%
	PKIS	30.0%	8.5%	5.9%	3.3%	33.3%	5.8%	3.5%	1.8%
	ksrMKL	40.0%	11.8%	7.8%	4.3%	76.7%	16.3%	7.9%	4.2%
Fyn	iGPS	46.4%	18.6%	15.0%	9.0%	57.1%	15.7%	10.4%	5.7%
	PKIS	21.4%	7.6%	7.2%	4.3%	39.3%	9.8%	7.2%	4.0%
	ksrMKL	46.4%	18.6%	15.0%	9.0%	62.5%	17.4%	11.3%	6.2%
ATM	iGPS	51.2%	32.5%	33.1%	24.5%	57.3%	25.6%	24.2%	15.4%
	PKIS	59.8%	37.8%	37.6%	27.5%	79.3%	36.6%	32.0%	20.1%
	ksrMKL	90.9%	55.8%	52.1%	36.5%	97.6%	45.4%	38.0%	23.6%
Lck	iGPS	65.2%	17.7%	9.9%	5.3%	65.2%	11.9%	5.3%	2.7%
	PKIS	30.4%	7.6%	4.7%	2.6%	47.8%	8.2%	3.9%	2.0%
	ksrMKL	69.6%	18.9%	10.5%	5.7%	91.3%	17.5%	7.3%	3.8%
MAPK1	iGPS	40.6%	26.6%	28.6%	22.1%	62.2%	29.3%	27.7%	17.8%
	PKIS	21.7%	13.1%	16.4%	13.1%	36.7%	15.4%	17.3%	11.3%
	ksrMKL	40.6%	26.6%	28.6%	22.1%	66.1%	31.3%	29.2%	18.7%
MAPK3	iGPS	39.9%	24.1%	25.3%	18.5%	47.3%	19.5%	19.0%	11.9%
	PKIS	20.3%	11.1%	13.7%	10.3%	34.5%	13.0%	14.2%	8.9%
	ksrMKL	41.2%	25.0%	26.0%	19.0%	64.2%	27.9%	24.9%	15.4%
SRC	iGPS	55.6%	41.8%	44.4%	37.0%	57.8%	31.7%	33.3%	23.4%
	PKIS	38.4%	29.2%	33.0%	28.9%	55.6%	30.3%	32.2%	22.7%
	ksrMKL	84.3%	60.6%	60.4%	47.1%	98.1%	54.7%	50.6%	34.1%
CDK1	iGPS	39.0%	31.1%	35.3%	32.3%	42.2%	23.2%	27.6%	20.5%
	PKIS	26.0%	20.2%	25.0%	24.1%	43.2%	23.9%	28.1%	20.9%
	ksrMKL	48.7%	38.6%	42.3%	37.3%	69.8%	40.8%	41.9%	29.9%
GSK3b	iGPS	12.6%	5.4%	8.3%	6.1%	18.5%	4.4%	7.3%	4.6%
	PKIS	17.0%	8.4%	11.0%	8.1%	32.6%	11.5%	12.6%	7.8%
	ksrMKL	31.9%	18.2%	19.6%	14.2%	45.9%	18.1%	17.3%	10.6%
MAPK14	iGPS	35.0%	17.9%	18.0%	12.1%	62.1%	22.9%	18.5%	10.9%
	PKIS	16.5%	7.1%	8.9%	6.1%	25.2%	6.9%	8.0%	4.7%
	ksrMKL	31.1%	15.7%	16.1%	10.9%	40.8%	13.8%	12.6%	7.4%
PKCa	iGPS	29.1%	21.7%	25.8%	23.3%	39.6%	20.2%	23.9%	17.1%
	PKIS	27.2%	20.1%	24.4%	22.1%	36.2%	18.0%	22.1%	15.9%
	ksrMKL	35.5%	26.8%	30.7%	27.0%	54.3%	29.4%	31.4%	22.1%
Average	iGPS	37.3%	18.7%	18.8%	14.8%	47.2%	16.3%	15.5%	10.3%
	PKIS	26.7%	13.4%	14.5%	11.8%	41.3%	14.2%	14.1%	9.5%
	ksrMKL	49.4%	25.9%	24.8%	18.9%	68.5%	25.7%	21.9%	14.4%

Average: Average performance of all kinases

Table S3. Information of top 20 potential phosphorylation sites for MAPK1 kinase.

Ranking	UniProt ID	Protein Name	Position	Score	Ranking	UniProt ID	Protein Name	Position	Score
**1**	P01100	FOS	331	1.100	**11**	Q15149	PLEC	4539	0.764
**2**	Q15796	SMAD2	220	0.997	**12**	P24928	POLR2A	1616	0.745
**3**	Q9UBK2	PPARGC1A	299	0.907	**13**	P01106	MYC	58	0.739
**4**	P10276	RARA	77	0.856	**14**	Q9UBK2	PPARGC1A	263	0.717
**5**	P17676	CEBPB	235	0.853	**15**	P36956	SREBF1	439	0.707
**6**	P56270	MAZ	72	0.841	**16**	P41212	ETV6	257	0.688
**7**	Q9NQU5	PAK6	165	0.829	**17**	P01100	FOS	325	0.683
**8**	P19634	SLC9A1	726	0.826	**18**	P49137	MAPKAPK2	25	0.658
**9**	P40763	STAT3	727	0.826	**19**	Q15796	SMAD2	245	0.648
**10**	Q04206	RELA	529	0.821	**20**	Q9UJU2	LEF1	155	0.628
