# Supplementary figures and images for: ksrMKL: a novel method for identification of kinase–substrate relationships using multiple kernel learning
Source: PeerJ. 2017 Dec 20;5:e4182. doi: 10.7717/peerj.4182 (PMC5741978; doi:10.7717/peerj.4182)

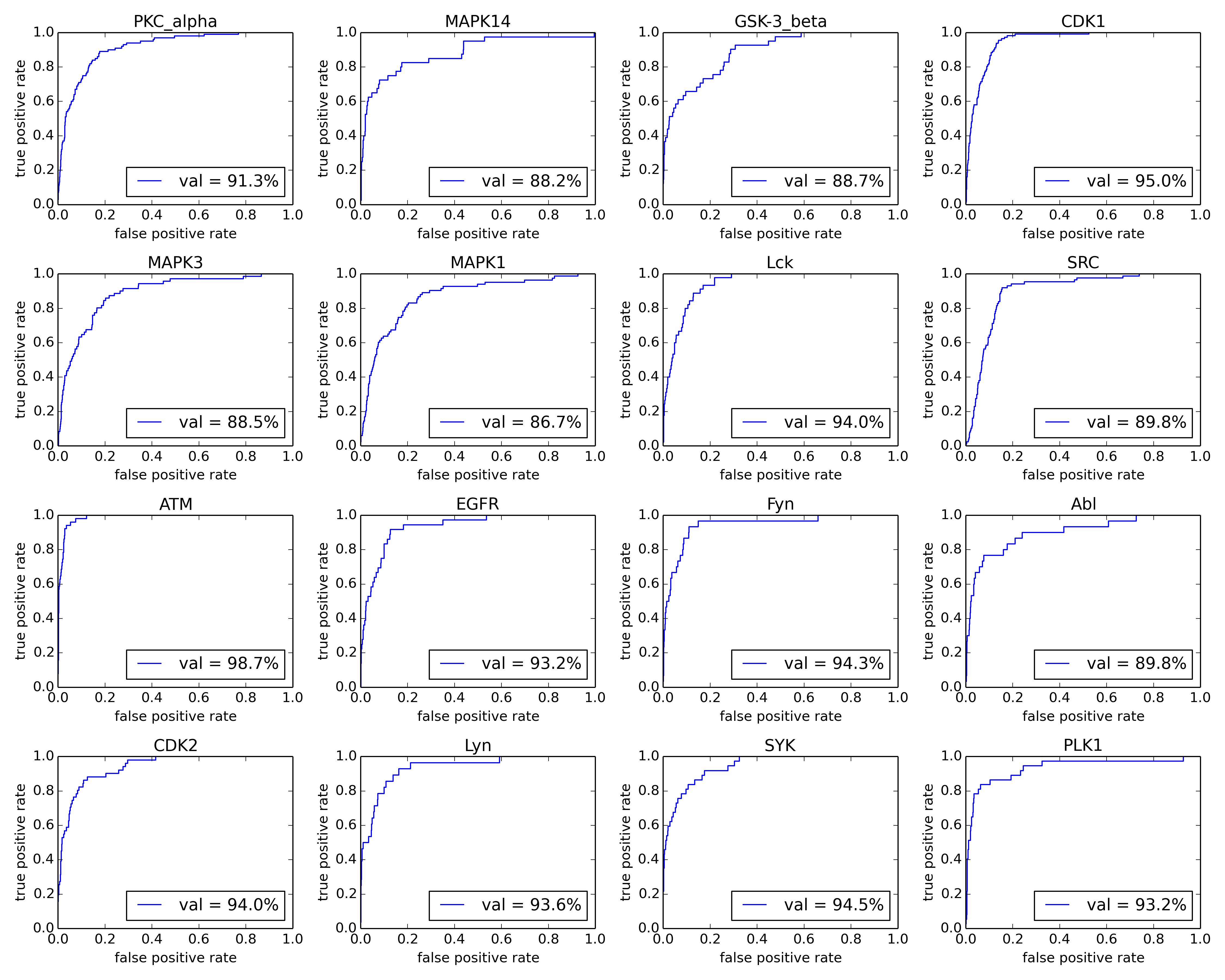

Supplement: Supplemental Information 2 [file peerj-05-4182-s002.zip › ksrMKL.png]
